# *Toxoplasma gondii*-infected natural killer cells display a hypermotility phenotype *in vivo*

**DOI:** 10.1038/icb.2014.106

**Published:** 2014-12-23

**Authors:** Norikiyo Ueno, Melissa B Lodoen, Graeme L Hickey, Ellen A Robey, Janine L Coombes

**Affiliations:** 1 Department of Molecular Biology and Biochemistry and The Institute for Immunology, University of California, Irvine, CA, USA; 2 Department of Epidemiology and Population Health, Institute of Infection and Global Health, University of Liverpool, Liverpool, UK; 3 Department of Molecular and Cell Biology, University of California, Berkeley, CA, USA; 4 Department of Infection Biology, Institute of Infection and Global Health, University of Liverpool, Liverpool, UK

## Abstract

*Toxoplasma gondii* is a highly prevalent intracellular protozoan parasite that causes severe disease in congenitally infected or immunocompromised hosts. *T. gondii* is capable of invading immune cells and it has been suggested that the parasite harnesses the migratory pathways of these cells to spread through the body. Although *in vitro* evidence suggests that the parasite further enhances its spread by inducing a hypermotility phenotype in parasitized immune cells, *in vivo* evidence for this phenomenon is scarce. Here we use a physiologically relevant oral model of *T. gondii* infection, in conjunction with two-photon laser scanning microscopy, to address this issue. We found that a small proportion of natural killer (NK) cells in mesenteric lymph nodes contained parasites. Compared with uninfected ‘bystander’ NK cells, these infected NK cells showed faster, more directed and more persistent migratory behavior. Consistent with this, infected NK cells showed impaired spreading and clustering of the integrin, LFA-1, when exposed to plated ligands. Our results provide the first evidence for a hypermigratory phenotype in *T. gondii*-infected NK cells *in vivo*, providing an anatomical context for understanding how the parasite manipulates immune cell motility to spread through the host.

Toxoplasmosis is a common zoonosis caused by the obligate intracellular protozoan parasite, *Toxoplasma gondii*. Initial infection occurs orally, but the parasite rapidly traverses tissues and biological barriers, disseminating widely through the host.

*T. gondii* is capable of invading any nucleated cell, including cells of the immune system.^1^ Immune cells are often highly motile and adept at traversing biological barriers and it is thought that *T. gondii* makes use of these existing properties to reach distant tissues.^2, 3, 4, 5^ For example, dendritic cells, CD11b^+^ cells and T cells have been shown to promote parasite dissemination *in vivo*.^2, 4, 6^ Furthermore, *in vitro* assays reveal that *T. gondii* actively manipulates the migratory patterns of the cells it invades. Infected myeloid cells become ‘hypermotile’, displaying rapid cytoskeletal rearrangement, impaired adhesion to extracellular matrix and increased chemotaxis.^2, 7, 8, 9, 10, 11, 12^ Alterations in monocyte rolling and transendothelial migration through endothelial barriers under shear stress have also recently been described.^13, 14^ These behavioral changes are often accompanied by changes in the expression, activation or clustering of integrins.^7, 13, 14, 15^ Athough these observations are suggestive of the manipulations in cell behavior that would allow *T. gondii* to travel through tissues and across barriers more easily, a ‘hypermotility’ phenotype in invaded cells has not yet been directly observed *in vivo*. Given the important role played by the tissue environment in regulating immune cell motility, a tractable *in vivo* assay will be crucial to understanding how *T. gondii* manipulates immune cell motility to enhance its spread.

Natural killer (NK) cells have a protective role in *T. gondii* infection, but are susceptible to direct invasion by the parasite.^16, 17, 18, 19, 20, 21, 22, 23^ We have recently shown that NK cells are recruited to foci of *T. gondii* infection in the subcapsular sinus of the lymph node, where their migration and localization are regulated by α2β1-integrin-mediated interactions with collagen.^17^ Here we demonstrate that *T. gondii* invades NK cells and alters their migration in lymph nodes, providing direct evidence for a *T. gondii-*induced immune cell hypermotility phenotype *in vivo*.

## Results

### *T. gondii*-infected NK cells display a hypermotility phenotype *in vivo*

Direct infection of immune cells by *T. gondii* results in a hypermotility phenotype in *in vitro* assays.^2, 8, 9, 11, 12, 13^ However, two-photon laser scanning microscopy analysis of T cells and neutrophils migrating in intact living tissues has shown that the motility of the parasitized cells does not differ significantly from their uninfected counterparts.^6, 24, 25^ We recently showed that NK cells accumulate in foci of *T. gondii* infection beneath the lymph node capsule.^17^ In these experiments, we consistently observed that a small proportion of these NK cells contained parasites. We therefore assessed the impact of direct invasion by *T. gondii* on NK cell behavior in intact, living tissues.

**Figure 1:**
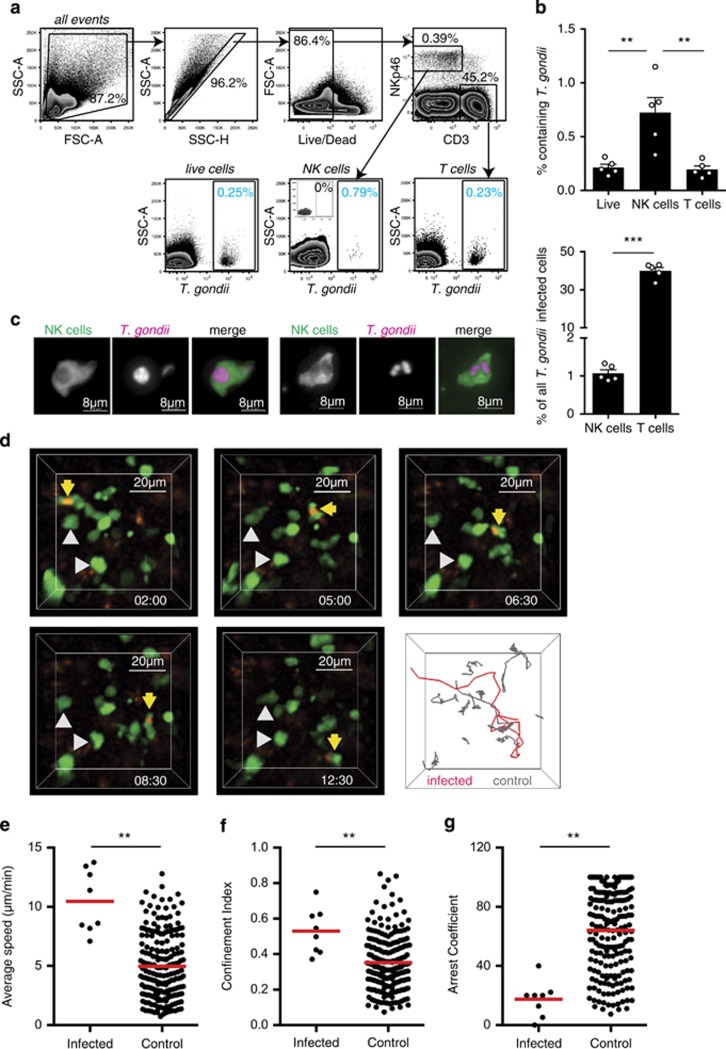
*T. gondii*-infected NK cells display a hypermotility phenotype *in vivo.* (**a**) Flow cytometric analysis of mesenteric lymph node at day 5 following oral infection is shown. Plots show gating of live, single cells into T-cell (CD3^+^) and NK cell (NKp46^+^CD3^−^) populations (top row). The percentage of cells in each population containing *T. gondii* is then determined by gating on parasite fluorescence (blue numbers, bottom row). The inset plot depicts an uninfected control sample. (**b**) Graphs show the percentage of the indicated cell population that contains *T. gondii* (mean±s.e.m. of five mice) and the percentage of *T. gondii*-infected cells that are T cells or NK cells. (**c**) Fluorescence microscopy of the mesenteric lymph node from an *Ncr1*^GFP/+^ mouse 6 days after oral infection is shown. NK cells are green, *T. gondii* is pink. (**d**) Individual time points and tracks from a two-photon laser scanning microscopy movie showing a *T. gondii-*infected NK cell migrating in the mesenteric lymph node 4 days after oral infection are shown. NK cells are green, *T. gondii* is red. An infected NK cell is highlighted with yellow arrows/red track and uninfected NK cells with gray arrows/tracks. Corresponds to Supplementary Movie 1. (**e**–**g**) Graphs show the average speed (**e**) confinement index (**f**) and arrest coefficient (**g**) of individual NK cells. For each condition data are pooled from five imaging volumes obtained over the course of three independent experiments (*n*=3, days 4–5 post infection). **P<0.001.

To detect and visualize NK cells, we used mice in which one copy of the *Ncr1* gene had been replaced with a green fluorescence protein (GFP) reporter.^26^ These mice were infected via the physiologically relevant oral route with tissue cysts of the type II *Prugniaud* strain engineered to express tdTomato, allowing us to monitor the infection levels in NK cells by flow cytometry.^6^ Five days after oral infection, 0.72±0.14% of NK cells in the draining mesenteric lymph nodes contained parasites (Figures 1a and b). This was greater than the proportion of T cells containing parasites (0.20±0.03%) or the proportion of infected cells in lymph node as a whole (0.21±0.03%, Figures 1a and b). Nevertheless, the relative abundance of T cells in the lymph node when compared with NK cells meant that they accounted for a high proportion of *T. gondii*-infected cells (Figure 1b).^6^ NK cells containing intact parasites could be readily visualized in mesenteric lymph node sections (Figure 1c). In some cases, multiple parasites were observed within a single NK cell (Figure 1c).

We then used two-photon laser scanning microscopy to compare the motility of *T. gondii*-infected and uninfected ‘bystander’ NK cells in the mesenteric lymph nodes of orally infected mice (Figure 1d,Supplementary Movie 1). Using a standard linear regression model, *T. gondii*-infected NK cells migrated 6.00 μm min^−1^ faster than noninfected cells after adjustment for differences between mice (95% confidence interval (CI): 4.10–7.90; *P*<0.001, Figure 1e). The linearity of the path taken by a cell can be described by the confinement index (maximum displacement/path length), where higher values indicate more linear migration. Using the linear regression model, the confinement index was 0.203 units greater in the *T. gondii*-infected cells (95% CI: 0.093–0.313; *P*<0.001, Figure 1f). The arrest coefficient is the percentage of time that a cell’s speed falls below 5 μm min^−1^ and is generally high when NK cells form stable contacts with target cells or immotile tissue structures. The arrest coefficient was smaller by an absolute value of 50.64 percentage points in the *T. gondii*-infected cells, indicating that stable contacts with immotile cells or structures in the lymph node were greatly reduced (95% CI: 31.63–69.66; *P*<0.001, Figure 1g). This faster, more directed and more persistent migratory behavior allows NK cells to cover more ground, potentiating the spread of the parasite.

### Infected NK cells display impaired cell spreading and integrin clustering.

NK cells use integrins to form low motility contacts with the extracellular matrix and target cells. For example, VLA-2 (CD49b:CD29, α2β1) mediates low motility contacts between NK cells and collagen fibers in the lymph node, whereas LFA-1 (CD11a:CD18, αLβ2) is involved in NK cell adhesion to, and killing of, target cells.^17, 27, 28, 29, 30, 31^ The increased motility of *T. gondii*-infected NK cells could therefore be explained by parasite-driven alterations in integrin expression or activity.

**Figure 2:**
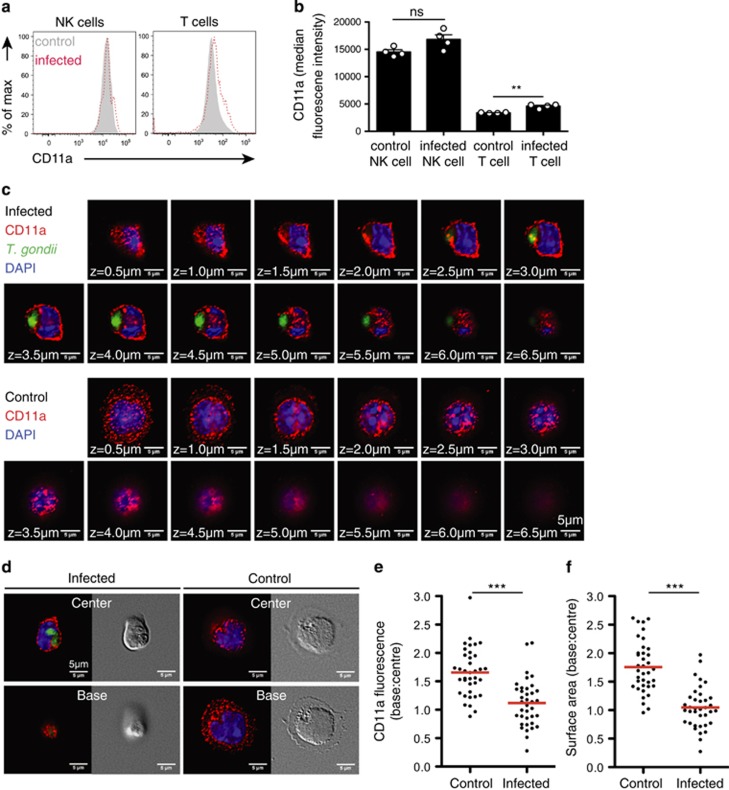
Infected NK cells display impaired integrin clustering and cell spreading. (**a**) Flow cytometric analysis of CD11a expression on NK cells in mesenteric lymph nodes at day 5 following oral infection is shown. Plots are derived from concatenated samples from four individual mice analyzed. Infected NK cells are shown in red and bystander NK cells in gray. (**b**) Graph shows the median fluorescence intensity of CD11a on the indicated cell populations (mean±s.e.m. of four mice). (**c** and **d**) Immunofluorescence analysis of CD11a distribution on the NK cell surface in response to ICAM-1 ligand. Uninfected and *T. gondii*-infected NK cells were settled onto immobilized mouse ICAM-1/Fc. After 15–30 mins, samples were fixed and stained to detect surface CD11a by fluorescence microscopy. *Z*-sections from the cell base to the cell top were acquired at intervals of 0.5 μm. Representative fluorescent and differential interference contrast micrographs from three independent experiments are shown. CD11a is shown in red, the parasites in green and the nuclei in blue. Corresponds to Supplementary Movie 2. (**e** and **f**) Differences in CD11a distribution and surface area between uninfected and infected cells were quantified as ratios of their respective values at the cell base to the cell center (*n*_uninfected_=37, *n*_infected_=37 cells, from three independent experiments). Red bars show the mean.

Integrin activity is regulated by conformational changes to the receptor and by dynamic alterations in expression, trafficking, clustering or distribution.^32^ Our initial experiments demonstrated that oral infection did not alter NK cell surface expression of a panel of integrins tested, including the CD11a subunit of LFA-1 (Figures 2a and b, Supplementary Figure 1).

To assess whether *T. gondii* infection alters integrin clustering, we infected NK cells with *T. gondii* and seeded the NK cells onto ICAM-1 coated cover glass.^13^ CD11a (LFA-1) localization was determined by confocal imaging of the NK cells from the point of contact with the ICAM-1-coated surface, to the top of the cell, at 0.5-μm intervals (Figure 2c). In uninfected NK cells, CD11a clustered in the contact zone between the NK cell and the ICAM-1-coated surface. However, in infected cells, CD11a was more evenly distributed over the entire surface of the cell (Figures 2c–e, Supplementary Movie 2). Furthermore, although uninfected cells showed evidence of cell spreading at the point of contact with the ligand, the infected cells were more rounded in morphology (Figure 2f).

Given the important role played by integrins in the formation of low motility contacts with target cells and the extracellular matrix, the observed reduction in cell spreading and redistribution of integrin in *T. gondii*-infected NK cells is consistent with the absence of low motility behavior we observe in these cells *in vivo*.

## Discussion

Infection with *T. gondii* has significant socioeconomic costs, both in terms of severe disease in the human population and economic losses in farming. Understanding how the parasite spreads through the host will be important in the design of novel vaccines and therapeutics aimed at minimizing the burden of infection in the brain or preventing transplacental transmission to the developing fetus. Here we used a physiologically relevant oral model of *T. gondii* infection to show that *T. gondii*-infected NK cells display a hypermotility phenotype *in vivo*. Our data provide (1) crucial support for the hypothesis that *T. gondii* manipulates immune cell motility to spread through its host and (2) a cellular and anatomical context to understand how the parasite achieves this in complex tissues.

Our data reveal that impaired cell spreading and CD11a/LFA-1 clustering in *T. gondii*-infected NK cells is a possible mechanism for their altered motility in tissues. Intermediate levels of integrin-mediated adhesion are usually optimal for cell migration, whereas too much or too little adhesion can negatively impact cell motility.^33^ Thus, the reduction in cell spreading and LFA-1 clustering observed in *T. gondii*-infected NK cells is consistent with the changes in motility observed *in vivo*, and implies that NK cells are constantly using LFA-1 to contact other cells or structures in tissues. Similarly, LFA-1 has been implicated in the intranodal migration of T cells, whereas *in vitro* studies have shown that LFA-1 triggers asymmetrical NK cell spreading and migration.^34, 35, 36^ Interestingly, enhanced transmigration was not observed in parasitized human NK cells migrating *in vitro*, suggesting that the anatomical context in which migration takes place is an important contributing factor to the hypermotility phenotype in NK cells.^8^ Infection of other immune cell populations by *T. gondii* has also been associated with changes in the expression, activation or clustering of integrins.^7, 13, 14, 15^ For example, *T. gondii*-infected macrophages display a reduction in adhesiveness to extracellular matrix components, which is accompanied by reduced surface expression of multiple integrins, including LFA-1^7^. Furthermore, *T. gondii*-infected human monocytes, which rolled at higher speeds and for longer distances over endothelial cells, displayed impaired LFA-1 clustering and cell spreading.^13^

Our results raise the possibility that hypermotile NK cells have an important role in facilitating the spread of *T. gondii* through the host. This idea is supported by an earlier study demonstrating that NK cells become infected following lytic contacts with infected dendritic cells, but are not susceptible to lysis by other NK cells.^23^ Although it has been shown that adoptive transfer of *T. gondii*-infected immune cells results in higher infection loads when compared with the inoculation with free parasites, an important question is whether hypermotility of endogenous NK cells, or other immune cells, contribute to the spread of infection in a natural setting.^2, 4^ This type of experiment is complicated by the protective roles that immune cells also play in infection. A better understanding of how *T. gondii* alters immune cell motility is therefore necessary to design experiments to address this question. Following initial infection in the small intestine, *T. gondii* spreads through the intestine, from the intestine to the lymph nodes and blood, and from the blood to the brain, to muscle, and across the placenta to the developing fetus. Different immune cell populations display distinct migratory pathways, and we favor the idea that the parasite utilizes different immune cell populations at different stages in this process. For example, neutrophils have been implicated in the luminal spread of parasites through the small intestine, preventing T cell egress from the lymph nodes reduces the spread of the parasite to the spleen, and CD11b^+^ cells are implicated in the delivery of parasites to the brain.^4, 6, 24^ Although the exact role of NK cells remains to be determined, the role of uterine NK cells in transplacental transmission of infection is of particular interest in this respect. The ability to directly visualize hypermotility in infected immune cells in an *in vivo* infection model will provide an important platform for these studies.

## Methods

### Mice

CBA/J mice were purchased from The Jackson Laboratory. *Ncr1*^*GFP/+*^ mice were a gift from Dr O. Mandelboim (The Hebrew University of Jerusalem).^26^ Mice were housed under specific pathogen-free conditions at the AALAC-approved animal facility in the University of California, Berkeley, CA, USA. Animal experiments were approved by the Animal Care and Use Committee of the University of California.

### *T. gondii* infections

Type II *Prugniaud* parasites engineered to express tdTomato and ovalbumin were used for oral infections.^6^ Brain homogenates were prepared from CBA/J mice infected intraperitoneally with 400 tachyzoites 3–6 weeks previously. Cysts were counted after staining with Dolichos Biflorus Agglutinin (Vector Laboratories, Burlingame, CA, USA) and 50 cysts were administered by gavage to *Ncr1*^*GFP/+*^ mice. For infections *in vitro*, type II *Prugniaud* parasites engineered to express GFP were used. Murine NK cells were enriched from the spleen of wild-type C57BL/6 mice by negative magnetic selection (Stemcell Technologies, Vancouver, BC, Canada). Tachyzoites were added to purified NK cells at a multiplicity of infection of 2, and the mixture was incubated for 3 h at 37 °C.

### Two-photon imaging

Two-photon imaging was performed on the mesenteric lymph nodes from *Ncr1*^*GFP/+*^ mice 4–5 days after oral infection. Lymph nodes were explanted and perfused in warmed oxygenated media, as previously described.^37^ Images were acquired using a custom-built microscope with a Spectra-Physics (Santa Clara, CA, USA) MaiTai laser (tuned to 920 nm) and a 20 × /0.95 Nikon objective (Nikon, Melville, NY, USA). Emission light was separated with 495 , 510 and/or 560 nm dichroics, and collected with photomultiplier tube detectors. To minimize spectral overlap a bandpass filter, (HQ 450/80 M) was used. In some cases, imaging data were subjected to post-acquisition processing to limit spectral cross talk or background signal. Nonspecific background signal was subtracted using a digital mask generated on an unrelated channel using Imaris software, and/or a Gaussian filter was applied. Any adjustments made to brightness or contrast were linear and applied to the whole image. The *x*, *y* and *z* coordinates of the NK cells were obtained with Imaris software (Bitplane, Zurich, Switzerland). Motility parameters were calculated with MATLAB (MathWorks, Natick, MA, USA). There is a partial overlap between the raw datasets (image files) used to compile Figures 1e–g in this paper and Figure 2e in reference 17.

### Fluorescence microscopy

Six days after oral infection with tdTomato-expressing parasites, the mesenteric lymph nodes from *Ncr1*^*GFP/+*^ mice were prepared for fluorescence microscopy as previously described.^16^ Images were acquired using a Nikon Eclipse TE2000-E. Immunofluorescence microscopy of surface integrins was performed as previously described.^13^ In brief, uninfected and *T. gondii*-infected NK cells were settled onto ICAM-1/Fc-coated cover glass. Cells were then fixed with paraformaldehyde and stained with a monoclonal antibody against mouse CD11a (M17/4, Biolegend, San Diego, CA, USA) and Alexafluor 594-conjugated anti-rat secondary antibody (Life Technologies, Carlsbad, CA, USA). Cover glasses were mounted onto slides with Vectashield with DAPI (Vector Labs, Burlingame, CA, USA) and imaged using the × 60 objective lens of a Nikon Eclipse Ti fluorescent microscope. Micrographs were analyzed using ImageJ software (National Institutes of Health, Bethesda, MD, USA) and the fluorescence intensities and cell surface areas were plotted using GraphPad Prism Software (Graphpad, La Jolla, CA, USA).

### Flow cytometry

Single-cell suspensions were prepared from mesenteric lymph nodes of *Ncr1*^*GFP/+*^ mice 5 days after oral infection with tdTomato-expressing parasites. Cells were stained with a fixable Aqua Live/Dead dye (Life Technologies), then with antibodies to mouse CD3ε (145-2C11, Ebioscience, San Diego, CA, USA) and CD11a (M17/4, Ebioscience). Data were acquired using a BD LSR II (BD Biosciences, San Jose, CA, USA) and analyzed with FlowJo software (Tree Star, Ashland, OR, USA).

### Statistics

Unless otherwise noted, values are expressed as mean±s.e.m. For Figure 1b, the levels of significance were calculated by one-way analysis of variance with Tukey's *post hoc* tests. For Figure 2e, the Student’s two-tailed *t*-test with Welch’s correction was used (both GraphPad Prism). For analysis of cell motility data, linear regression models were fit to each observed motility parameter, with adjustment for mouse and a binary indicator of whether the cell was infected with *T. gondii* or not. The adjustment for mouse accounts for heterogeneity between mice, although this is not of interest. The estimated model coefficient for infection is interpreted as the difference in motility parameter between an infected cell and noninfected cell. The normality of fitted model residuals were visually inspected using quantile–quantile plots and a suitable transformation applied to the motility data where appropriate. For confinement index, inspection of the residuals suggested a log-transformation was appropriate to satisfy normality assumptions. After adjustment for variation in individual mice, the difference in log-confinement index between *T. gondii*-infected and noninfected cells was 0.607 (95% CI: 0.286–0.929; *P*<0.001). As the arrest coefficient is a percentage, the use of linear regression methods is a limitation. A Mann–Whitney *U*-test comparing the arrest coefficient between infected and noninfected cells (after pooling data from all mice) also confirmed a statistically significant difference (*P*<0.001). The Mann–Whitney *U*-test also yielded a significant difference for speed (*P*=0.002) and confinement (*P*<0.001) Regression analyses were done using the R statistical computing language version 3.0.2 (R Foundation for Statistical Computing, Vienna, Austria, 2013). Differences were considered significant at *P*<0.05. and are indicated with an asterisk (*P<0.05, **P<0.001, ***P<0.0001). 'NS' is not significant.
