# Supplementary figures and images for: Toxoplasma gondii-infected natural killer cells display a hypermotility phenotype in vivo
Source: Immunol Cell Biol. 2014 Dec 23;93(5):508–13. doi: 10.1038/icb.2014.106 (PMC4446200; doi:10.1038/icb.2014.106)

Supplementary Figure 1

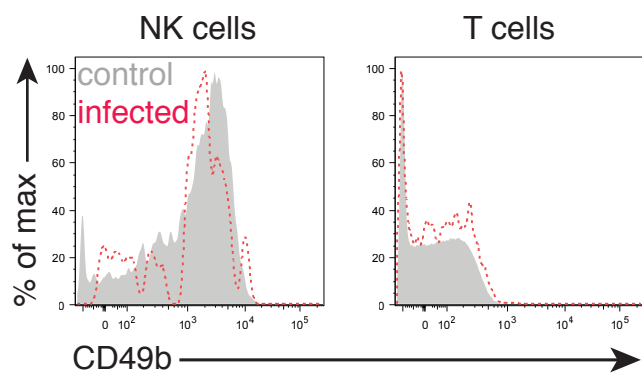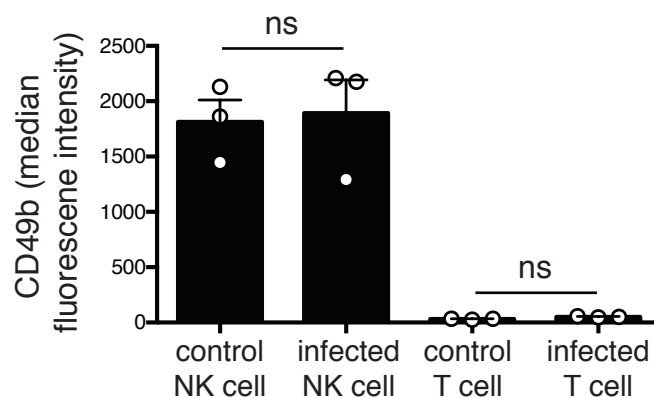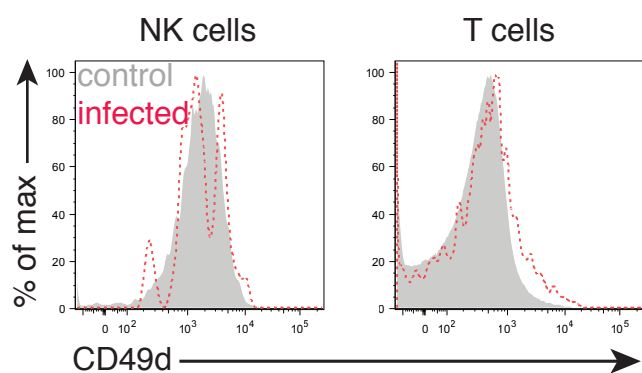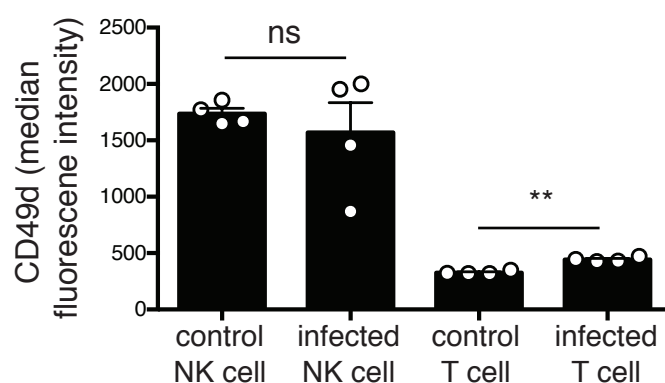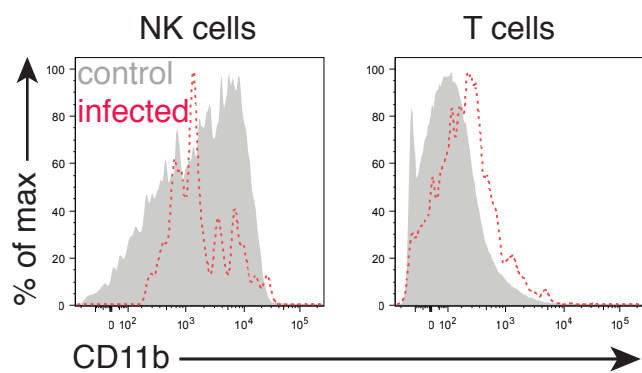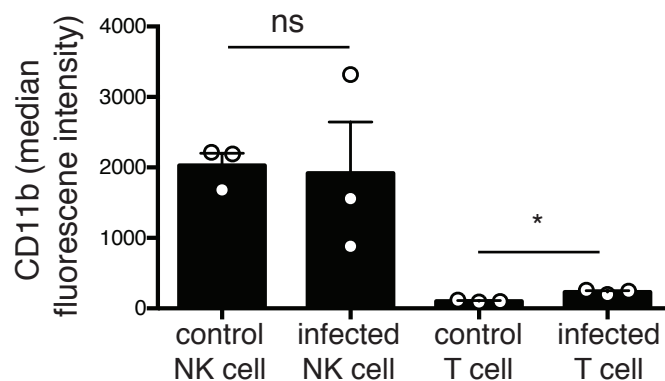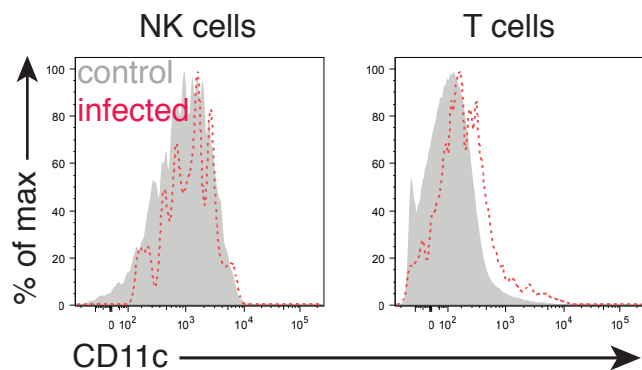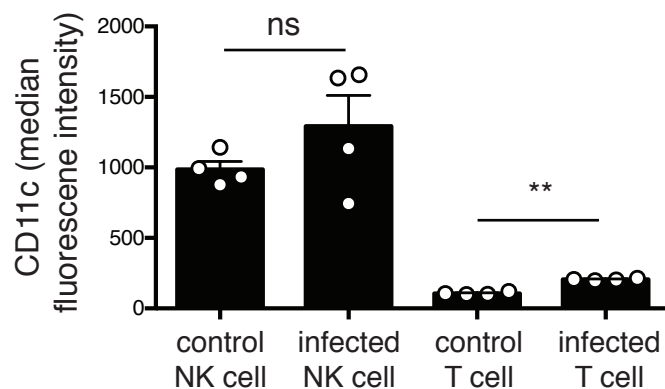

Supplement: Supplementary Figure 1 [file icb2014106x1.pdf]
