# Supplementary material for: Toxoplasma gondii-infected natural killer cells display a hypermotility phenotype in vivo
Source: Immunol Cell Biol. 2014 Dec 23;93(5):508–13. doi: 10.1038/icb.2014.106 (PMC4446200; doi:10.1038/icb.2014.106)
Supplement: Supplementary Information [file icb2014106x4.docx]

**Supplementary Figure 1**. (a) Flow cytometric analysis of integrin expression on NK cells in mesenteric lymph nodes at day 5 following oral infection is shown. Plots are derived from concatenated samples from 3-4 individual mice analysed. Infected NK cells are shown in red, and bystander NK cells in grey. (b) Graphs shows the median fluorescence intensity of integrin expression on the indicated cell populations (mean ± SEM of 3-4 mice).

**Supplementary Movie 1.**TPLSM of mesenteric lymph node 4 days after oral infection with *T. gondii*. NK cells are shown in green, and *T. gondii* in red. The tracks of uninfected NK cells are shown in white, and of a parasitized NK cell in red. Corresponds to Figure 1d.

**Supplementary Movie 2.** 3-D reconstruction of surface CD11a on a *T. gondii*-infected NK cell (right) and an adjacent uninfected bystander NK cell (left) adhered to immobilized ICAM-1. CD11a is shown in red, the parasites in green, and the nuclei in blue. Corresponds to Figure 2c-f.
